# Alcohol Intake Is Associated With Elevated Serum Levels of Selenium and Selenoprotein P in Humans

**DOI:** 10.3389/fnut.2021.633703

**Published:** 2021-02-22

**Authors:** Yuki Isobe, Hiroki Asakura, Hiromasa Tsujiguchi, Takayuki Kannon, Hiroaki Takayama, Yumie Takeshita, Kiyo-aki Ishii, Takehiro Kanamori, Akinori Hara, Tatsuya Yamashita, Atsushi Tajima, Shuichi Kaneko, Hiroyuki Nakamura, Toshinari Takamura

**Affiliations:** ^1^Department of Endocrinology and Metabolism, Kanazawa University Graduate School of Medical Sciences, Kanazawa, Japan; ^2^Department of Environmental and Preventive Medicine, Kanazawa University Graduate School of Medical Sciences, Kanazawa, Japan; ^3^Department of Bioinformatics and Genomics, Kanazawa University Graduate School of Medical Sciences, Kanazawa, Japan; ^4^Department of Gastroenterology, Kanazawa University Graduate School of Medical Sciences, Kanazawa, Japan

**Keywords:** alcohol, selenium, selenoprotein P, diabetes, fatty liver, hepatokine

## Abstract

Selenoprotein P is a hepatokine with antioxidative properties that eliminate a physiologic burst of reactive oxygen species required for intracellular signal transduction. Serum levels of selenoprotein P are elevated during aging and in people with type 2 diabetes, non-alcoholic fatty liver disease, and hepatitis C. However, how serum levels of full-length selenoprotein P are regulated largely remains unknown, especially in the general population. To understand the significance of serum selenoprotein P levels in the general population, we evaluated intrinsic and environmental factors associated with serum levels of full-length selenoprotein P in 1,183 subjects participating in the Shika-health checkup cohort. Serum levels of selenium were positively correlated with liver enzymes and alcohol intake and negatively correlated with body mass index. Serum levels of selenoprotein P were positively correlated with age, liver enzymes, and alcohol intake. In multiple regression analyses, alcohol intake was positively correlated with serum levels of both selenium and selenoprotein P independently of age, gender, liver enzymes, and fatty liver on ultrasonography. In conclusion, alcohol intake is associated with elevated serum levels of selenium and selenoprotein P independently of liver enzyme levels and liver fat in the general population. Moderate alcohol intake may exert beneficial or harmful effects on health, at least partly by upregulating selenoprotein P. These findings increase our understanding of alcohol-mediated redox regulation and form the basis for the adoption of appropriate drinking guidelines.

## Introduction

Selenoprotein P (encoded by the *SELENOP* gene in humans) is a secretory protein that contains multiple selenocysteine residues per polypeptide and functions as a selenium transport protein (1). Selenoprotein P exerts antioxidative properties directly via an N-terminal thioredoxin domain and indirectly by supplying selenium to antioxidative glutathione peroxidases (2). We have re-discovered selenoprotein P as a hepatokine that causes multi-signal resistances leading to type 2 diabetes, such as insulin resistance (3), angiogenesis resistance (4), ischemia-reperfusion injury (5), insulin secretory failure (6), and exercise resistance (7). Selenoprotein P eliminates a physiologic burst of reactive oxygen species required for intracellular signal transduction, a condition referred to as “reductive stress” (2). Serum levels of selenoprotein P are elevated during aging (8), and in people with type 2 diabetes (3), non-alcoholic fatty liver disease (9), and chronic hepatitis C (10).

The full-length selenoprotein P is digested with plasma kallikrein, which generates N-terminal and C-terminal fragments of selenoprotein P (11). Indeed, the selenium content of selenoprotein P was reported to be 5.4 ± 0.5 (mean ± SD) in humans (12), which seems lower than its theoretical ten selenocysteine residues per polypeptide. Therefore, we previously developed a sol particle homogeneous immunoassay method that selectively measures a full-length form of selenoprotein P in human serum (13, 14). Using this assay system, we reported selenium and full-length selenoprotein P status in a general Japanese population as follows: (1) serum levels of selenium and selenoprotein P are significantly correlated with each other; (2) both serum levels of selenium and selenoprotein P increase with aging; (3) serum levels of selenoprotein P, but not those of selenium, correlated positively with glucose levels and negatively with initial insulin secretion capacity in oral glucose tolerance tests; and (4) elevated serum levels of selenoprotein P, but not those of selenium, predict future onset of hyperglycemia (8). These findings suggest that serum levels of selenoprotein P and selenium, to some extent, serve as surrogate markers for redox and health status in humans.

Selenium supply and its incorporation into selenocysteine upregulate the *SELENOP* gene expression (1). Besides, several transcription factors are involved in *SELENOP* expression. Insulin downregulates *SELENOP* by phosphorylating and inactivating FoxO1 (15), whereas antidiabetic metformin activates AMP-activated protein kinase (AMPK) and phosphorylates and inactivates FoxO3a, subsequently downregulating *SELENOP* in hepatocytes (16). Eicosapentaenoic acid, one of the ω-3 polyunsaturated fatty acids, downregulates *SELENOP* by inhibiting nuclear transport and promoter binding of SREBP-1c (17). Hepatitis C viral infection upregulates *SELENOP* in the liver through C/EBPα (10). However, the regulation of serum levels of full-length selenoprotein P is poorly understood, especially in the general population.

In the present study, we aim to assess the intrinsic and environmental factors associated with serum levels of selenium and full-length selenoprotein P in the general population. We found that moderate alcohol intake is associated with serum levels of selenium and selenoprotein P regardless of liver injury.

## Materials and Methods

### Study Population

We used cross-sectional data from participants in the “Shika study” project of 2013–2017, conducted in the Noto Peninsula, Ishikawa, Japan, since 2011. The Shika study is an ongoing population-based survey that seeks to develop advanced preventive methods for lifestyle-related diseases. It includes interviews, self-administered questionnaires, and comprehensive health examinations. Shika town is located in a rural area of the Ishikawa prefecture, Japan. The town has over 20,000 residents (18). Data were collected from adults above 40 years of age in the model districts. All subjects with no reported gender (*n* = 8) were excluded from the study. All subjects gave their written informed consent for inclusion in the study. The study was conducted following the Declaration of Helsinki, and the protocol was approved by the Ethics Committee of Kanazawa University (No.1491).

### Measurements

Age, sex, height, weight, waist circumference, and systolic and diastolic blood pressure (SBP and DBP) were measured at health checkups for all participants. Body mass index (BMI) was calculated as weight in kilograms divided by the square of height in meters.

### Nutrient Assessment

We assessed alcohol intake from the validated food frequency questionnaire. We used the brief-type self-administered diet history questionnaire (BDHQ) developed in Japan for large-scale nutritional epidemiology studies (19). BDHQ asks for the dietary history for fifty-eight food items taken in the preceding month. These food items are commonly consumed in Japan and are mainly from the food list used in the National Health and Nutrition Survey of Japan. Participants who reported an energy intake of <600 kcal/day (half of the required energy for the lowest physical activity category) or more than 4,000 kcal/day (1.5 times the energy intake required for the highest physical activity category) were excluded from the analyses because they were either extremely low or extremely high energy intakes.

### Alcohol Consumption

Alcohol consumption was estimated using the BDHQ (19). The BDHQ asks for the consumption frequency and amount of Japanese sake, beer, wine, whiskey, and brandy, commonly consumed in Japan. Estimated alcohol consumption was calculated using an *ad hoc* computer algorithm, which included weighting factors for BDHQ. Alcohol intake levels were stratified as 0, <30, and ≥30 (g/day) in men and 0, <20, and ≥20 (g/day) in women (Supplementary Table 1).

### Smoking

Smoking status was stratified as non/past-smokers and current smokers.

### Assessment of Fatty Liver

Hepatic steatosis was determined using B-mode ultrasonography performed by experienced hepatologists. The presence of hepatic steatosis was determined by at least one of the following findings: increased hepatorenal contrast, liver brightness, deep attenuation, and vascular blurring. The severity of fatty liver was semi-quantitatively graded as mild (liver brightness or hepatorenal contrast), moderate (mild plus deep attenuation or vascular blurring), and severe (all four findings) (20).

### Blood Collection and Assays

Fasting blood samples were collected between 0800 and 1,200 h from the forearm vein of each participant. The serum samples were delivered to Kanazawa University through a commercial laboratory (SRL Kanazawa Laboratory, Kanazawa, Japan). The sera were frozen and stored at −30°C until the assay. Serum concentrations of full-length selenoprotein P were specifically measured by sol particle homogeneous immunoassay using two monoclonal antibodies, as previously established (13, 14). Serum concentrations of selenium were measured by atomic absorption spectrophotometry (8).

### Statistical Analyses

Normally distributed data were presented as means ± standard deviations, and the differences between the two groups were analyzed using the Student's *t*-test; the paired *t*-test was used for paired samples. Non-normally distributed data were presented as medians and ranges, and the differences between these groups were assessed using the Mann–Whitney *U*-test. Relationships were determined using regression analyses, and a *P* < 0.05 was considered statistically significant. Multivariate logistic regression analyses (forced entry method) were performed using age, gender, and liver enzymes as explanatory factors and selenoprotein P/selenium as dependent variables. All the explanatory variables were tested for collinearity, and only those that were confirmed to have no collinearity using the values of variance inflation factor and tolerance were used as independent explanatory variables in the multiple regression analyses. A two-way ANCOVA was performed to compare mean selenoprotein P/selenium levels and alcohol intake levels among participants with different liver enzyme levels. A simple main effect test was performed for selenoprotein P/selenium levels, in which interactions were assessed. All statistical analyses were conducted using SPSS software, version 16.0 (IBM, Armonk, NY, USA).

## Results

### Participant Characteristics

A total of 1,183 subjects (551 men) were included in the analyses. Of these, 525 (349 men) had a drinking habit, and the average amount of alcohol consumption was 12.96 g/day (30.17 g/day for men and 10.13 g/day for women) (Table 1). There were no significant differences in BMI, selenium, selenoprotein P, HbA1c, and fasting plasma glucose between genders or between drinkers and non-drinkers. In men, drinkers had significantly lower BMI than non-drinkers (*P* = 0.019). Insulin levels were not different between drinkers and non-drinkers and were significantly higher in women than in men (*P* < 0.001, data not shown).

**Table 1:** Characteristics of the study participants.

	**All**		**Men**				**Women**			
			**No drinking**		**Drinking**		**No drinking**		**Drinking**	
	***N***	**Mean ± S.D.**	***N***	**Mean ± S.D.**	***N***	**Mean ± S.D.**	***N***	**Mean ± S.D.**	***N***	**Mean ± S.D.**
Age (year)	1,183	62.1 ± 11.2	98	64.3 ± 12.0	349	61.6 ± 10.8	327	65.1 ± 11.2	176	58.1 ± 10.5
BMI (kg/m^2^)	1,181	23.3 ± 3.3	98	24.5 ± 3.6	349	23.8 ± 3.0	326	23.0 ± 3.3	175	22.2 ± 3.1
Selenium (μg/L)	752	158.74 ± 27.98	77	154.27 ± 24.73	273	165.48 ± 25.28	251	153.65 ± 32.37	134	155.99 ± 13.38
Selenoprotein P (μg/mL)	948	3.93 ± 0.90	98	3.83 ± 0.62	336	4.16 ± 0.85	318	3.76 ± 1.01	173	3.83 ± 3.1
Aspartate aminotransferase (U/L)	1,181	24.21 ± 10.456	98	24.24 ± 9.62	348	26.93 ± 14.66	326	22.02 ± 6.61	176	22.78 ± 1.84
Alanine aminotransferase (U/L)	1,181	22.29 ± 13.633	98	27.68 ± 21.48	348	25.92 ± 15.68	326	18.35 ± 8.87	176	19.66 ± 1.44
γ-glutamyl transpeptidase (IU/L)	1,181	41.63 ± 53.997	98	36.35 ± 33.95	348	68.33 ± 79.40	326	23.81 ± 22.13	176	30.84 ± 17.95
Energy intake (kcal/day)	950	1864.54 ± 632.58	98	1874.76 ± 587.14	349	2154.92 ± 652.04	327	1658.22 ± 568.01	176	1666.36 ± 3.4
Alcohol intake (g/day)	950	12.96 ± 21.64	98	–	349	30.17 ± 25.89	327	–	176	10.13 ± 3.0
Alcohol intake (% energy)	950	4.50 ± 7.14	98	–	349	10.04 ± 8.12	327	–	176	4.37 ± 3.0
HbA1c (%)	1,182	5.91 ± 0.66	98	6.24 ± 0.91	348	5.88 ± 0.68	327	5.92 ± 0.59	176	5.77 ± 3.5
Fasting plasma glucose (mg/dL)	1,068	96.42 ± 18.229	90	101.92 ± 25.58	314	99.35 ± 19.88	295	93.65 ± 14.66	160	92.24 ± 14.48
Insulin (μU/mL)	1,140	5.73 ± 4.90	92	6.05 ± 5.37	334	5.53 ± 5.77	313	5.77 ± 4.03	169	5.02 ± 3.0

### Factors Associated With Serum Selenium Levels

In all participants, selenium levels were correlated positively with AST, ALT, γGTP, and alcohol intake and negatively with BMI (Table 2a).

**Table 2a:** Univariate correlation between clinical parameters and selenium in participants consuming and not consuming alcohol.

	**Selenium**																	
	**All**						**Men**						**Women**					
			**No drinking**		**Drinking**				**No drinking**		**Drinking**				**No drinking**		**Drinking**	
	**R**	***p***	**R**	***p***	**R**	***p***	**R**	***p***	**R**	***p***	**R**	***p***	**R**	***p***	**R**	***p***	**R**	***p***
Age (year)	−0.043	0.244	−0.028	0.609	0.009	0.861	−0.157	0.003	−0.096	0.405	−0.151	0.013	0.050	0.327	−0.011	0.863	0.262	0.002
BMI (kg/m^2^)	−0.079	0.031	−0.135	0.014	−0.027	0.583	−0.063	0.230	−0.046	0.691	−0.061	0.318	−0.148	0.003	−0.163	0.010	−0.109	0.208
Smoking	0.126	<0.001	0.061	0.275	0.122	0.014	0.138	0.009	0.174	0.131	0.118	0.052	0.009	0.862	0.015	0.808	−0.023	0.792
Log AST	0.112	0.002	0.073	0.187	0.115	0.020	0.063	0.237	0.033	0.775	0.048	0.426	0.101	0.046	0.086	0.174	0.155	0.073
Log ALT	0.122	<0.001	0.091	0.099	0.108	0.029	0.064	0.223	0.124	0.282	0.042	0.489	0.094	0.063	0.086	0.174	0.119	0.172
log γGTP	0.249	<0.001	0.129	0.019	0.281	<0.001	0.286	<0.001	0.164	0.155	0.269	<0.001	0.121	0.016	0.126	0.046	0.118	0.175
Energy intake (kcal/day)	0.017	0.648	−0.064	0.251	0.027	0.592	−0.039	0.468	−0.273	0.016	−0.022	0.718	−0.034	0.501	−0.014	0.825	−0.095	0.275
Alcohol intake (g/day)	0.228	<0.001	–	–	0.262	<0.001	0.269	<0.001	–	–	0.238	<0.001	0.078	0.126	–	–	0.147	0.091
Alcohol intake (%energy)	0.234	<0.001	–	–	0.271	<0.001	0.283	<0.001	–	–	0.255	<0.001	0.082	0.109	–	–	0.156	0.072

*AST, aspartate aminotransferase; ALT, alanine aminotransferase; γGTP, γ-glutamyl transpeptidase*.

**Table 2b:** Univariate correlation between clinical parameters and selenoprotein P in participants consuming and not consuming alcohol.

	**Selenoprotein P**																	
	**All**						**Men**						**Women**					
			**No drinking**		**Drinking**				**No drinking**		**Drinking**				**No drinking**		**Drinking**	
	**R**	***p***	**R**	***p***	**R**	***p***	**R**	***p***	**R**	***p***	**R**	***p***	**R**	***p***	**R**	***p***	**R**	***p***
Age (year)	0.099	0.002	0.095	0.054	0.167	<0.001	0.030	0.528	−0.016	0.874	0.064	0.243	0.160	<0.001	0.121	0.031	0.300	<0.001
BMI (kg/m^2^)	−0.032	0.333	−0.084	0.086	0.022	0.618	−0.010	0.839	0.037	0.715	0.002	0.970	−0.106	0.018	−0.121	0.031	−0.070	0.360
Smoking	0.158	<0.001	0.142	0.004	0.124	0.005	0.154	0.001	0.24	0.017	0.118	0.031	0.067	0.137	0.133	0.017	−0.018	0.819
Log AST	0.136	<0.001	0.059	0.232	0.167	<0.001	0.128	0.007	0.075	0.464	0.126	0.021	0.085	0.057	0.054	0.337	0.157	0.040
Log ALT	0.095	0.003	0.040	0.419	0.104	0.019	0.073	0.123	0.141	0.167	0.069	0.207	0.024	0.596	0.005	0.923	0.051	0.504
Log γGTP	0.197	<0.001	0.103	0.035	0.188	<0.001	0.196	<0.001	0.174	0.087	0.154	0.005	0.079	0.076	0.085	0.128	0.053	0.491
Energy intake (kcal/day)	0.057	0.085	−0.019	0.702	0.060	0.178	0.008	0.869	−0.190	0.062	0.008	0.889	−0.006	0.894	0.007	0.898	−0.041	0.593
Alcohol intake (g/day)	0.212	<0.001	–	–	0.221	<0.001	0.237	<0.001	–	–	0.193	<0.001	0.058	0.200	–	–	0.091	0.232
Alcohol intake (%energy)	0.212	<0.001	–	–	0.219	<0.001	0.244	<0.001	–	–	0.201	<0.001	0.058	0.200	–	–	0.091	0.232

*AST, aspartate aminotransferase; ALT, alanine aminotransferase; γGTP, γ-glutamyl transpeptidase*.

In men, selenium levels were correlated positively with GGT and alcohol intake and negatively with age in the drinkers, whereas they were correlated negatively with energy intake in the non-drinkers. In women, selenium levels were correlated positively with age in the drinkers, whereas they were correlated positively with γGTP and negatively with BMI in the non-drinkers (Table 2a).

**Figure 1:**
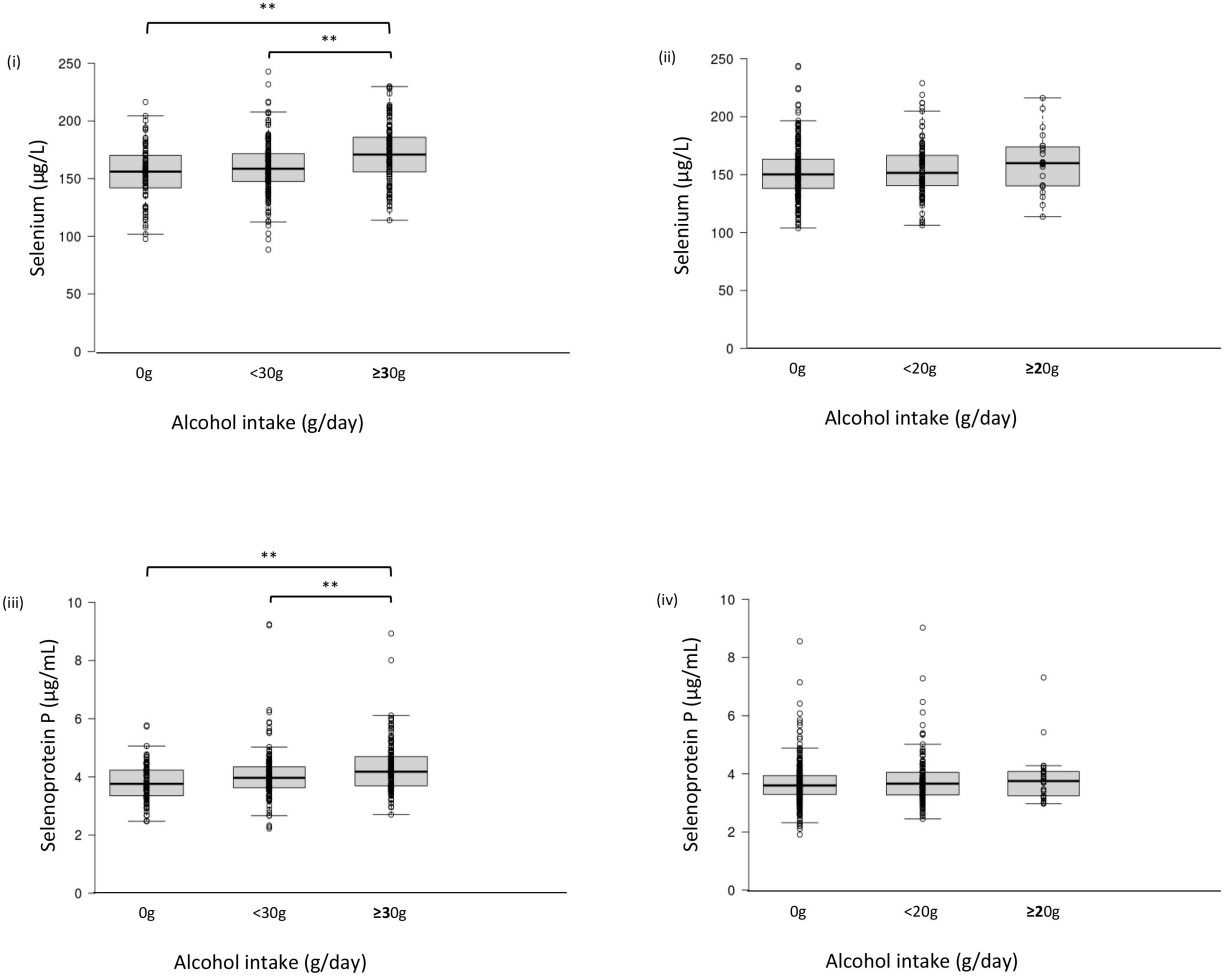
Serum selenium/selenoprotein P levels in each group of alcohol intake. Serum level of selenium (i, ii) and selenoprotein P (iii, iv) in men (i, iii) and women (ii, iv). In box-plots, center lines show the medians, and box limits indicate the 25th and 75th percentiles; whiskers extend 1.5x the interquartile range from the 25th and 75th percentiles; data points are plotted as dots. **p <0.01.

**Figure 2:**
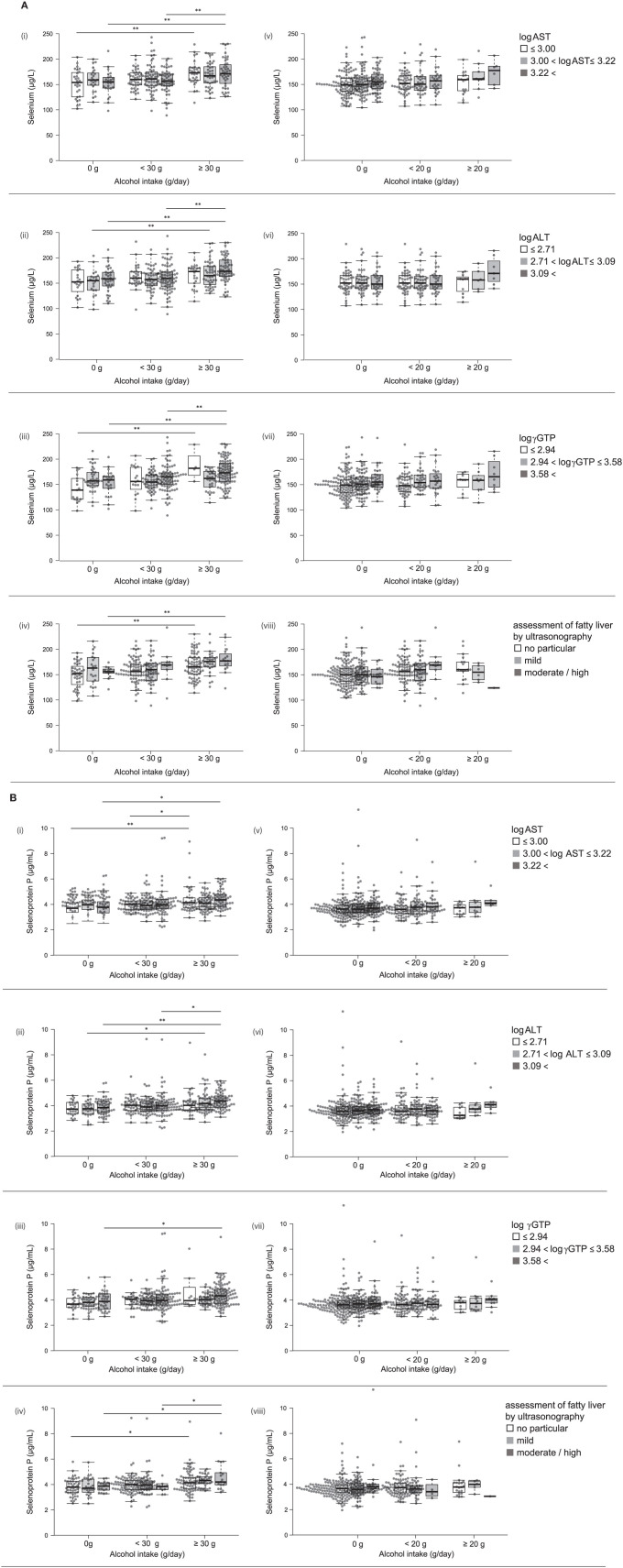
**(A)** Serum levels of selenium and selenoprotein P in participants with different alcohol intake levels and different liver enzyme levels. Serum levels of selenium in participants according to various levels of log AST (i, v), log ALT (ii, vi), log γGTP (iii, vii), and fatty liver by ultrasonography (iv, viii) in men (i~iv) and women (v~viii). **(B)** Serum levels of selenoprotein P in participants with different alcohol intake levels and different liver enzyme levels. Serum levels of selenoprotein P in participants according to various levels of log AST (i, v), log ALT (ii, vi), log γGTP (iii, vii), and fatty liver by ultrasonography (iv, viii) in men (i~iv) and women (v~viii). In box-plots, center lines show the medians, and box limits indicate the 25th and 75th percentiles; whiskers extend 1.5x the interquartile range from the 25th and 75th percentiles; data points are plotted as dots. *p < 0.05, **p < 0.01.

Serum selenium levels in men were increased by alcohol intake in a dose-dependent manner (Figure 1-i) but not in women (Figure 1-ii). In multiple regression analyses (Table 3a, Figure 2A), alcohol intake was positively correlated with serum levels of selenium independently of age, gender, liver enzymes, and fatty liver on ultrasonography in both men and women. Furthermore, in men, similar results were obtained with an adjustment for smoking (Table 3a). In women, however, the correlation between alcohol intake and selenium disappeared with a smoking adjustment (Table 3a).

**Table 3a:** Relationship between alcohol intake and selenium in participants taking alcohol.

		**Selenium**								
		**All**			**Men**			**Women**		
		**β**	**t**	***p***	**β**	**t**	***p***	**β**	**t**	***p***
Alcohol intake (g/day)	Adjusted for age, gender, and log AST	0.220	4.262	<0.001	0.235	3.987	<0.001	0.200	2.370	0.019
	Adjusted for age, gender, and log ALT	0.228	4.431	<0.001	0.239	4.094	<0.001	0.201	2.389	0.018
	Adjusted for age, gender, and log γGTP	0.172	3.256	0.001	0.176	2.941	0.004	0.184	2.113	0.037
	Adjusted for age, gender, and assessment of fatty liver by ultrasonography	0.239	4.461	<0.001	0.248	4.087	<0.001	0.212	2.436	0.016
										
Alcohol intake (g/day)	Adjusted for age, gender, smoking, and log AST	0.179	4.351	<0.001	0.246	4.686	<0.001	0.094	1.713	0.088
	Adjusted for age, gender, smoking, and log ALT	0.189	4.626	<0.001	0.252	4.871	<0.001	0.100	1.825	0.069
	Adjusted for age, gender, smoking, and log γGTP	0.136	3.189	0.001	0.178	3.255	0.001	0.080	1.453	0.147
	Adjusted for age, gender, smoking, and assessment of fatty liver by ultrasonography	0.196	4.617	<0.001	0.262	4.899	<0.001	0.106	1.836	0.067

*AST, aspartate aminotransferase; ALT, alanine aminotransferase; γGTP, γ-glutamyl transpeptidase*.

**Table 3b:** Relationship between alcohol intake and selenoprotein P inparticipants taking alcohol.

		**Selenoprotein P**								
		**All**			**Men**			**Women**		
		**β**	**t**	***p***	**β**	**t**	***p***	**β**	**t**	***p***
Alcohol intake (g/day)	Adjusted for age, gender, and log AST	0.174	3.742	<0.001	0.181	3.333	<0.001	0.147	2.001	0.047
	Adjusted for age, gender, and log ALT	0.184	3.965	<0.001	0.191	3.560	<0.001	0.148	2.014	0.046
	Adjusted for age, gender, and log γGTP	0.162	3.361	<0.001	0.163	2.898	0.004	0.146	1.944	0.054
	Adjusted for age, gender, and assessment of fatty liver by ultrasonography	0.196	3.943	<0.001	0.205	3.509	<0.001	0.162	2.077	0.040
										
Alcohol intake (g/day)	Adjusted for age, gender, smoking, and log AST	0.155	4.260	<0.001	0.204	4.295	<0.001	0.079	1.666	0.096
	Adjusted for age, gender, smoking, and log ALT	0.166	4.604	<0.001	0.220	4.702	<0.001	0.081	1.729	0.084
	Adjusted for age, gender, smoking, and log γGTP	0.138	3.664	<0.001	0.179	3.594	<0.001	0.072	1.524	0.128
	Adjusted for age, gender, smoking, and assessment of fatty liver by ultrasonography	0.170	4.405	<0.001	0.228	4.551	<0.001	0.085	1.675	0.095

*AST, aspartate aminotransferase; ALT, alanine aminotransferase; γGTP, γ-glutamyl transpeptidase*.

We compared selenium levels among participants' alcohol intake levels and different liver enzyme levels using a two-way ANCOVA (Table 4a). The results showed a significant main effect of alcohol consumption on selenium levels only in men. A significant interaction was observed between alcohol intake levels and log γGTP levels for selenium.

**Table 4a:** Comparison of serum selenium levels (μg/L) among participants with different alcohol intake levels and different liver enzyme/fatty liver levels.

		**Men**							**Women**						
		**Alcohol intake**			***p*** **-value**				**Alcohol intake**			***p*** **-value**			
		**0 g**	** <30 g**	**≥30 g**	**Between alcohol intake levels**	**Between liver enzyme levels**	**Interaction between alcohol intake levels and liver enzyme levels**	**Between alcohol intake levels in each liver enzyme levels**	**0 g**	** <20 g**	**≥20 g**	**Between alcohol intake levels**	**Between lilver enzyme levels**	**Interaction between alcohol intake levels and liver enzyme levels**	**Between alcohol intake levels in each liver enzyme levels**
Log AST	≤ 3.00	151.214	160.690	172.533	<0.001	0.710	0.330	–	149.769	153.226	151.000	0.360	0.064	0.691	–
	3.00–3.22	159.174	163.789	168.282				–	153.250	156.281	165.875				–
	3.22 <	153.231	156.192	174.132				–	161.909	157.240	177.750				–
Log ALT	≤ 2.71	152.600	163.310	165.864	<0.001	0.241	0.452	–	151.227	153.951	151.273	0.540	0.083	0.561	–
	2.71–3.09	151.000	159.348	168.386				–	151.425	155.711	159.500				–
	3.09 <	157.111	159.750	176.964				–	162.167	155.613	175.571				–
LogγGTP	≤ 2.94	141.235	157.563	187.250	0.001	0.232	0.006	0.002	149.734	150.981	156.125	0.781	0.210	0.873	–
	2.94–3.58	160.703	156.080	158.885				0.670	158.418	159.514	154.750				–
	3.58 <	153.565	163.318	174.870				<0.001	159.966	157.348	170.375				–
Fatty liver	no particular	148.925	158.714	167.426	<0.001	0.039	0.466	–	152.488	154.871	164.706	0.895	0.324	0.786	–
	mild	161.250	158.467	173.846				–	152.827	152.786	154.333				–
	mederate / severe	154.538	168.600	179.667				–	147.769	151.500	124.000				–

**Table 4b:** Comparison of serum selenoprotein P levels (μg/mL) among participants with different alcohol intake leves and different liver enzyme/fatty liver levels.

		**Men**							**Women**						
		**Alcohol intake**			***p*** **-value**				**Alcohol intake**			***p*** **-value**			
		**0 g**	** <30 g**	**≥30 g**	**Between alcohol intake levels**	**Between liver enzyme levels**	**Interaction between alcohol intake levels and liver enzyme levels**	**Between alcohol intake levels in each liver enzyme levels**	**0 g**	** <20 g**	**≥20 g**	**Between alcohol intake levels**	**Between lilver enzyme levels**	**Interaction between alcohol intake levels and liver enzyme levels**	**Between alcohol intake levels in each liver enzyme levels**
Log AST	≤ 3.00	3.753	3.978	4.417	<0.001	0.477	0.317	–	3.670	3.689	3.620	0.529	0.072	0.851	–
	3.00–3.22	3.920	4.022	4.090				–	3.809	3.943	3.991				–
	3.22 <	3.847	4.100	4.431				–	3.872	3.907	4.362				–
Log ALT	≤ 2.71	3.784	4.027	4.184	<0.001	0.350	0.863	–	3.795	3.822	3.485	0.660	0.376	0.367	–
	2.71–3.09	3.709	4.037	4.291				–	3.720	3.891	4.088				–
	3.09 <	3.920	4.045	4.401				–	3.768	3.709	4.194				–
LogγGTP	≤ 2.94	3.740	3.949	4.586	0.001	0.087	0.465	–	3.706	3.763	3.646	0.949	0.377	0.899	–
	2.94–3.58	3.827	3.953	4.025				–	3.797	3.895	3.994				–
	3.58 <	3.901	4.115	4.390				–	3.955	3.807	4.003				–
Fatty liver	No particular	3.823	4.006	4.230	<0.001	0.819	0.412	–	3.766	3.771	3.952	0.903	0.661	0.754	–
	Mild	3.842	4.074	4.295				–	3.705	3.888	3.917				–
	Mederate/Severe	3.844	3.781	4.624				–	3.882	3.525	3.050				–

### Factors Associated With Serum Selenoprotein P Levels

In all participants, serum levels of selenoprotein P were positively correlated with age, AST, ALT, γGTP, and alcohol intake (Table 2b). In men, selenoprotein P levels were correlated positively with AST, γGTP, and alcohol intake in the drinkers. In women, selenoprotein P levels were correlated positively with age and AST in the drinkers, whereas they were correlated positively with age and negatively with BMI in the non-drinkers (Table 2b).

Among drinkers, alcohol intake higher than 30 g/day increased serum levels of selenoprotein P in men (Figure 1-iii) but not in women (Figure 1-iv). In multiple regression analyses (Table 3b, Figure 2B), alcohol intake was positively correlated with serum levels of selenoprotein P independently of age, gender, liver enzymes, and fatty liver on ultrasonography in both men and women. Furthermore, in men, similar results were obtained after adjusting for smoking. In women, however, the correlation between alcohol intake and selenoprotein P disappeared with a smoking adjustment (Table 3b).

We compared selenoprotein P among participants' alcohol intake levels at different liver enzyme levels using a two-way ANCOVA (Table 4b). The results showed a significant main effect of alcohol consumption on the selenoprotein P levels only in men. No significant interaction was observed between alcohol intake levels and liver enzyme levels for selenoprotein P.

### Intake of Macro/Micronutrients and Foods Associated With Serum Levels of Selenium or Selenoprotein P

Alcohol intake was associated with an estimated intake of total energy, protein, carbohydrate, protein ratio to total energy, zinc, copper, and manganese (Supplementary Table 2). However, selenium levels were not associated with these nutrients, and selenoprotein P levels were associated only with protein ratio to the total energy.

Alcohol intake was associated with relatively selenium-rich foods, such as seafood, especially in men (Supplementary Table 3).

## Discussion

In the present study, we found that moderate alcohol intake was associated with elevated serum levels of selenium and selenoprotein P independently of liver enzyme levels and liver fat in the general population. This finding provides the first evidence of a positive correlation between alcohol intake and serum levels of selenoprotein P in the general population.

Too much alcohol consumption should be avoided due to its health hazard aspects (21). Since any alcohol use is associated with short-term and long-term health risks, it seems difficult to define universally applicable population-based thresholds for low-risk drinking (22). Therefore, the WHO aims to prevent and reduce the harmful use of alcohol as a public health priority (21). On the other hand, alcohol intake has both positive and negative effects on health. It remains unresolved whether alcohol intake elevates or reduces oxidative stress (23, 24). Accumulating evidence suggests that alcohol consumption can elevate or reduce cardiovascular risk, depending on the dosage (25–27). Selenium and selenoprotein P also exert beneficial or adverse health effects on the development of diabetes (28) and cardiovascular diseases (29) depending on their concentrations and circumstances. Selenoprotein P is a crucial redox protein in the body, but in excess, it induces reductive stress leading to various forms of intracellular signal resistance, such as resistance to insulin, VEGF, and exercise (2). Therefore, selenoprotein P may interfere with alcohol-induced alterations in oxidative stress and mediate the known alcohol-mediated deleterious effects on health by impairing vasculogenesis (25, 26) and exercise performance (30).

To date, the reported effects of alcohol intake on selenium and selenoprotein P appear to be inconsistent. Contrary to the present findings, mounting evidence suggests that binge alcohol consumption or alcohol abuse is associated with lower serum selenium levels and downregulated selenoproteins in humans (31–34) and animals (35, 36). Lower selenium levels may cause the reduced selenoprotein P levels observed in cases of alcoholism. At least in rats, binge alcohol consumption reduces selenium absorption and downregulates hepatic expression of selenoproteins, such as *Gpx1* and *Gpx4* (encoding glutathione peroxidases one and four), but not *SELENOP* (36).

Reduced hepatic reserve caused by alcoholic hepatitis/cirrhosis may cause low serum levels of selenium (34). Among the selenoprotein family members, selenoprotein P is a primary source of selenium in the plasma as systemic removal of selenoprotein P reduces plasma levels of selenium to <10% (37). Although most of the organs produce selenoprotein P ubiquitously, the liver produces most of the selenoprotein P found in plasma (38). Therefore, the liver is most responsible for circulating selenium levels. We observed that hepatic expression of *SELENOP* significantly declines in the pre-cirrhotic/cirrhotic (F3/4) liver than in the F1/2 liver in patients with chronic hepatitis C (10). We speculate that impaired production of selenoprotein P in the cirrhotic liver reduces circulating levels of selenoprotein P.

Healthy cohort studies evaluating selenium and selenoprotein P are limited. Rasmussen et al. reported that fish intake correlated weakly with serum levels of selenium but not with those of selenoprotein P in 817 randomly selected subjects from two cities in Denmark (39). Smoking status, alcohol intake, exercise habits, BMI, and medicine use did not influence selenium status in their cohort. In a study of selenium status in 391 healthy residents in the south of England (40), daily drinkers showed lower plasma selenium concentrations than non-drinkers and weekend drinkers in men but not in women. In the 966 subjects with colorectal cancer and 966 matched control subjects participating in the European prospective investigation of cancer and nutrition cohort, alcohol intake is not associated with serum levels of selenium and selenoprotein P (41). These studies investigate European people whose blood levels of selenium and selenoprotein P are relatively low compared with the present study. Also, our study evaluated the full-length selenoprotein P levels selectively. These may affect the inconsistent findings between the present study and the previous European studies. On the other hand, our findings are consistent with the study investigated in 124 male and female subjects living in the States, half of whom consumed alcoholic beverages lightly or moderately (42). In that study, alcohol consumption is positively correlated with selenium level and GPx activity in the plasma and whole blood cells. In support of these findings, experimental data in rats has shown that alcohol consumption raises selenium levels in the liver and whole blood without influencing selenium absorption or retention (43). Put together, we can conclude that moderate alcohol intake in healthy individuals raises serum selenium and selenoprotein P levels. In contrast, binge alcohol consumption and alcoholic liver cirrhosis cause reduced serum selenium and selenoprotein P.

Selenium sources are derived from the diet. Also, *SELENOP* gene expression is regulated with macronutrients and hormones, positively with glucose and saturated fatty acids, and negatively with insulin (3). Thus, we investigated the association between intake of alcohol, macro/macronutrients, and foods and serum levels of selenium or selenoprotein P (Supplementary Tables 2, 3). Alcohol intake was associated with an estimated intake of total energy, protein, and carbohydrate (Supplementary Table 2). However, selenium/selenoprotein P levels were not closely associated with these nutrients, suggesting that nutritional alterations associated with alcohol intake seem unlikely to be involved in the elevated levels of selenium and selenoprotein P.

Selenium is abundant in seafood, animal organs, and eggs, followed by cereals (depending on soil selenium content), meat, and dairy products (Standard tables of food composition in Japan 2015–seventh revised version). In the present study, alcohol intake was positively associated with an intake of fish, squid, octopus, shrimp, shellfish, meat, egg, whole milk, and wheat (noodles, bread), which are abundant in selenium (Supplementary Table 3). These findings suggest that moderate alcohol consumption elevates serum levels of selenium and selenoprotein P via preferential intake of selenium-rich foods such as seafood.

In this study, alcohol intake is associated with elevated serum levels of selenium and selenoprotein P more strongly in men than women. One of the causes for such sexual dimorphic findings may be less alcohol intake in women than men (Supplementary Table 1, Figures 1, 2A,B). There were only 29 women with alcohol intake ≥20 g/day, whereas 155 men with alcohol intake ≥30 g/day (Supplementary Table 1). Even in men, alcohol intake over 30 g/day exerted apparent effects on selenium and selenoprotein P (Figures 1, 2A,B). Besides, alcohol intake was more frequently associated with an intake of selenium-rich food, such as seafood, in men than women (Supplementary Table 3), which may also be one of the causes for the sexual dimorphic findings.

It may be necessary to investigate the molecular mechanisms underlying alcohol intake-induced elevation of serum selenoprotein P levels. Besides selenium status, other environmental factors that affect transcription factor networks determine selenoprotein P levels (2). To date, various ethanol-responsive genes or altered genes in alcoholic hepatitis, including transcription factors, have been identified (44) and may be responsible for alcohol-induced *SELENOP* expression in the liver. Of note, ethanol treatment enhances the nuclear translocation of FoxO3a and upregulates the expression of FoxO3a-target genes in primary hepatocytes and mouse liver (45). Another study showed that alcohol administration downregulates FoxO1 but slightly upregulates FoxO3a in the liver of mice (46). We previously found that metformin activates AMPK and inhibits FoxO3a activity, thereby downregulating *SELENOP* in hepatocytes (16). Therefore, it may be possible that alcohol intake upregulates *SELENOP* expression by activating FoxO3a in the liver, which should be confirmed experimentally in the future.

This study has a limitation that alcohol intake was assessed by self-report, which may not be purely true and accurate.

In conclusion, alcohol intake is associated with elevated serum levels of selenium and selenoprotein P independently of liver enzyme level and liver fat in the general population, especially in men. Therefore, moderate alcohol intake may exert beneficial or adverse effects on health, at least partly by upregulating selenium and selenoprotein P. These findings increase our understanding of alcohol-mediated redox regulation, which leads to increased antioxidative capacity and increased risk for diabetes via reductive stress. The findings should also form the basis for creating appropriate drinking guidelines.

## Data Availability Statement

The datasets presented in this article are not readily available because several studies are ongoing using the datasets in the present study. Requests to access the datasets should be directed to ttakamura@med.kanazawa-u.ac.jp.

## Ethics Statement

The studies involving human participants were reviewed and approved by The Ethics Committee of Kanazawa University (No.1491). The patients/participants provided their written informed consent to participate in this study.

## Author Contributions

TT designed the study, interpreted the data, and wrote the manuscript with input from all authors. YI analyzed the data with supports by HA, HTs, AH, and HN. TKann and AT built the database for the cohort. HTa, YT, K-aI, TKana, and TT collected the data. TY and SK evaluated the hepatic steatosis. TT is the guarantor of this work and takes responsibility for the integrity of the data and accuracy of the data analysis. YI analyzed the data with supports by HA, HTs, AH, SK, and HN. All authors have read the manuscript and took part in the discussion.

## Conflict of Interest

The authors declare that the research was conducted in the absence of any commercial or financial relationships that could be construed as a potential conflict of interest.
