# Corrigendum: Alcohol Intake Is Associated With Elevated Serum Levels of Selenium and Selenoprotein P in Humans

**DOI:** 10.3389/fnut.2021.696947

**Published:** 2021-08-26

**Authors:** Yuki Isobe, Hiroki Asakura, Hiromasa Tsujiguchi, Takayuki Kannon, Hiroaki Takayama, Yumie Takeshita, Kiyo-aki Ishii, Takehiro Kanamori, Akinori Hara, Tatsuya Yamashita, Atsushi Tajima, Shuichi Kaneko, Hiroyuki Nakamura, Toshinari Takamura

**Affiliations:** ^1^Department of Endocrinology and Metabolism, Kanazawa University Graduate School of Medical Sciences, Kanazawa, Japan; ^2^Department of Environmental and Preventive Medicine, Kanazawa University Graduate School of Medical Sciences, Kanazawa, Japan; ^3^Department of Bioinformatics and Genomics, Kanazawa University Graduate School of Medical Sciences, Kanazawa, Japan; ^4^Department of Gastroenterology, Kanazawa University Graduate School of Medical Sciences, Kanazawa, Japan

**Keywords:** alcohol, selenium, selenoprotein P, diabetes, fatty liver, hepatokine

In the original article, there was a mistake in the box colors for Figure 2A and Figure 2B as published. **The colors of the boxes, from left to right, are light gray, medium gray, and dark gray, respectively**. The correct legend appears below.

**Figure 2:**
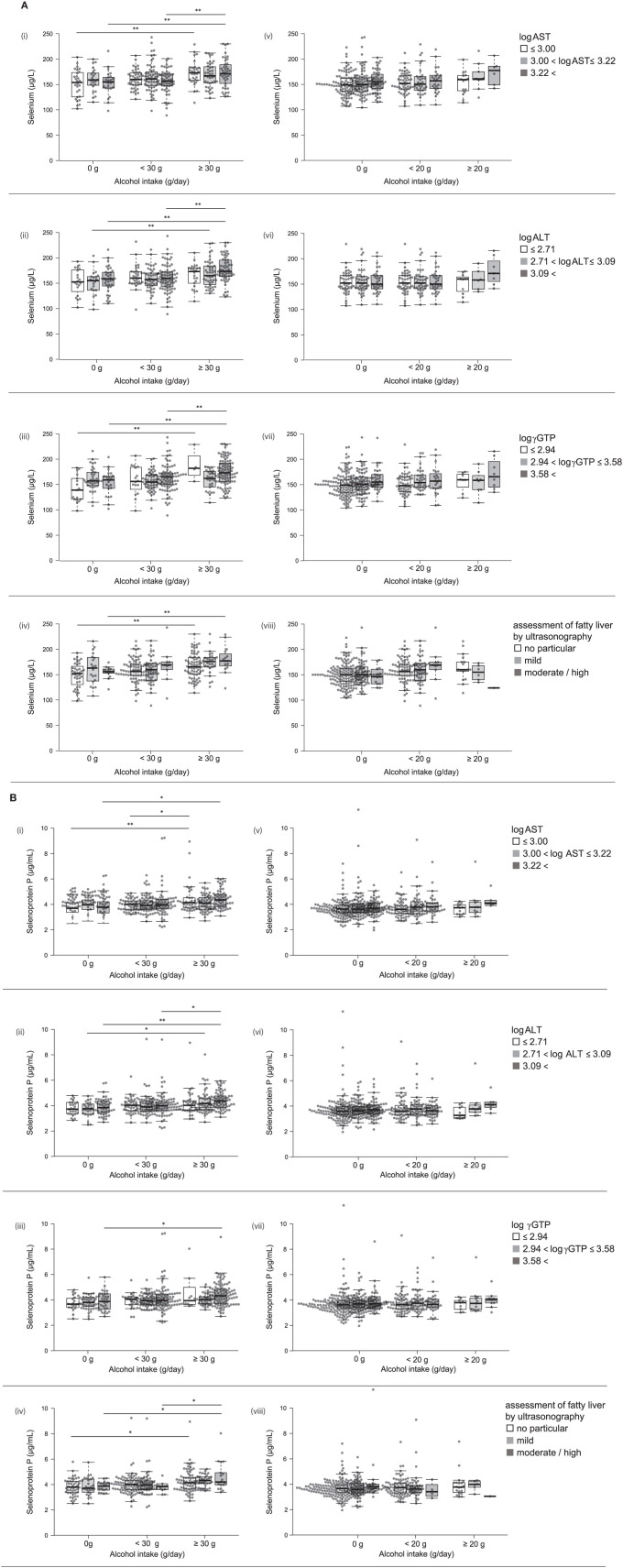
**(A)** Serum levels of selenium and selenoprotein P in participants with different alcohol intake levels and different liver enzyme levels. Serum levels of selenium in participants according to various levels of log AST (i,v), log ALT (ii,vi), log γGTP (iii,vii), and fatty liver by ultrasonography (iv,viii) in men (i~iv) and women (v~viii). **(B)** Serum levels of selenoprotein P in participants with different alcohol intake levels and different liver enzyme levels. Serum levels of selenoprotein P in participants according to various levels of log AST (i,v), log ALT (ii,vi), log γGTP (iii,vii), and fatty liver by ultrasonography (iv,viii) in men (i~iv) and women (v~viii). In box-plots, center lines show the medians, and box limits indicate the 25th and 75th percentiles; whiskers extend 1.5x the interquartile range from the 25th and 75th percentiles; data points are plotted as dots. *p < 0.05; **p < 0.01.

The authors apologize for this error and state that this does not change the scientific conclusions of the article in any way. The original article has been updated.

