# Supplementary material for: Alcohol Intake Is Associated With Elevated Serum Levels of Selenium and Selenoprotein P in Humans
Source: Front Nutr. 2021 Feb 22;8:633703. doi: 10.3389/fnut.2021.633703 (PMC7937717; doi:10.3389/fnut.2021.633703)
Supplement: Supplementary file 1 [file Table_1.docx]

Supplementary Table 1. Stratification of alcohol intake in men and women.

Supplementary Table 2. Univariate correlation between alcohol intake, serum levels of selenium and selenoprotein P, and intake of macronutrients and macronutrients.

Supplementary Table 3. Univariate correlation between intake of alcohol and selenium-rich foods.
